# Identification of hypoxia-related gene signatures and molecular subtypes in chronic rhinosinusitis with nasal polyps

**DOI:** 10.1016/j.bjorl.2026.101788

**Published:** 2026-03-19

**Authors:** Lulu Song, Shican Zhou, Lili Wang, Ju Lai, Shiwang Tan, Chunyan Yao, Shaoqing Yu

**Affiliations:** a Tongji University, School of Medicine, Tongji Hospital, Department of Otorhinolaryngology-Head and Neck Surgery, Shanghai, China; b Tongji University, School of Medicine, Tongji Hospital, Department of Allergy, Shanghai, China; c Tongji University, Tongji Hospital, Department of Stomatology, Shanghai, China

**Keywords:** Chronic rhinosinusitis, Nasal polyps, Hypoxia, Bioinformatics

## Abstract

**Objectives:**

Chronic Rhinosinusitis with Nasal Polyps (CRSwNP) is a common and severe subtype of Chronic Rhinosinusitis (CRS), characterized by persistent inflammation and tissue remodeling in the nasal cavity. Hypoxia, resulting from chronic inflammation, is believed to play a pivotal role in CRSwNP pathogenesis. This study aims to identify Hypoxia-Related Genes (HRGs) involved in CRSwNP and explore their potential as diagnostic biomarkers and therapeutic targets.

**Methods:**

We retrieved transcriptomic datasets (GSE136825 and GSE179265) from the Gene Expression Omnibus (GEO) database. Differential expression analysis was conducted to identify Hypoxia-Related Differentially Expressed Genes (HRG-DEGs) in CRSwNP. Machine learning techniques, including Least Absolute Shrinkage and Selection Operator (LASSO) regression and random forest, were employed to identify key HRGs. Validation of these genes was performed using qRT-PCR on clinical CRSwNP and control tissue samples. Additionally, consensus clustering analysis was applied to categorize CRSwNP patients into different molecular subtypes based on HRG expression, followed by gene ontology (GO) enrichment and immune infiltration analysis.

**Results:**

A total of 19 hypoxia-related Differentially Expressed Genes (DEGs) were identified. Through LASSO regression and Random Forest analysis, followed by validation using an external dataset and qRT-PCR of clinical samples, four hypoxia-related hub genes ‒ CXCR4, HMOX1, DTNA, and FBP1 ‒ were identified. Receiver Operating Characteristic (ROC) curve analysis demonstrated that all four genes had Area Under the Curve (AUC) values exceeding 0.8. Additionally, consensus clustering revealed two distinct clusters with different hypoxia characteristics.

**Conclusion:**

This study identifies four key hypoxia-related genes (CXCR4, HMOX1, DTNA, and FBP1) that may serve as novel diagnostic biomarkers for CRSwNP. The molecular subtypes identified through clustering provide further insights into CRSwNP heterogeneity, suggesting the need for personalized treatment approaches targeting hypoxia-related pathways.

## Highlights

• This study identifies four genes as potential biomarkers for CRSwNP.
• Machine learning techniques were used to effectively identify key genes.
• Clustering analysis reveals two distinct molecular subtypes of CRSwNP.
• This study identified sensitive drugs with therapeutic potential for two clusters.

## Introduction

CRS is one of the most prevalent chronic medical conditions worldwide that impacts individuals across all age. The incidence rates are substantial, with estimates suggesting 11.9% in the United States, 10.9% in Europe, and 8% in China.^1^, ^2^, ^3^ Furthermore, CRS imposes a considerable economic burden on society.^4^

CRS is characterized by a persistent inflammatory response within the nasal cavity, manifesting as excessive mucus production and nasal obstruction.^5^ CRS is classified into two distinct clinical phenotypes: CRS with Nasal Polyps (CRSwNP) and CRS without Nasal Polyps (CRSsNP).^6^ CRSwNP is often regarded as a more severe manifestation of the disease due to its propensity for recurrence.^7^ The primary therapeutic interventions for CRSwNP include glucocorticosteroids and surgical intervention; however, it has been found that many patients do not respond well.^8^ Therefore, there is a pressing requirement to delve into the pathogenesis of CRS to elucidate its etiology and, consequently, to develop efficient management approaches.

Previous studies have indicated that the development of CRS is related to pathological mechanisms induced by hypoxia. Specifically, in cases of CRSwNP, the nasal mucosa is subjected to hypoxia as a consequence of persistent inflammation and the mechanical compression of the local blood vessels by the nasal polyps.^9^^,^^10^ Moreover, hypoxia has been shown to impact the differentiation of nasal epithelial cells, including ciliated and goblet cells, and to stimulate the proliferation of fibroblasts. These effects can culminate in Epithelial-Mesenchymal Transition (EMT) and subsequent tissue remodeling.^11^, ^12^, ^13^, ^14^ However, comprehensive analysis of HRGs in bulk sequencing datasets has not been conducted. This study aims to address this gap by analyzing hypoxia-related genes within bulk sequencing data, providing a deeper understanding of their contributions to the pathogenesis of CRSwNP and potentially identifying novel therapeutic targets.

## Methods

### Bulk RNA sequencing (RNA-seq) data collection

The transcriptome data were obtained from the Gene Expression Omnibus (GEO) database, which can be accessed at https://www.ncbi.nlm.nih.gov/geo/. Two datasets were retrieved, including GSE136825 and GSE179265 dataset. GSE136825 dataset consisted of 28 samples of inferior turbinate from healthy control subjects and 42 samples of nasal polyp from CRSwNP patients. GSE179265 dataset included 7 samples of normal uncinate process and 17 samples of nasal polyp from CRSwNP patients.

### Differential expression genes (DEGs) analysis

The “DESeq” package was utilized to analyze differential expression in genetic profiles, aiming to investigate the molecular mechanisms underlying CRSwNP. DEGs were identified between control samples and CRSwNP samples, as well as among CRSwNP subtypes clustered based on the expression of HRGs. Only genes with a p-value < 0.05 and |log2 fold change| > 1.2 were selected for further analysis. The “pheatmap” and “ggplot2” *R*-packages were employed to construct heatmaps and volcano plots of the DEGs, respectively.

### Calculation of hypoxia scores using GSVA

The “GSVA” package was employed to calculate the hypoxia scores between CRSwNP and Control on the expression levels of hypoxia-related gene sets. The resulting hypoxia response scores were then visually represented using boxplots.

### Gene set enrichment analysis

The Gene Ontology (GO) and Kyoto Encyclopedia of Genes and Genomes (KEGG) pathway enrichment analyses were conducted using the enrichGO and enrichKEGG functions from the “clusterProfiler” package. Gene Set Enrichment Analysis (GSEA) was utilized to investigate pathway discrepancies among different groups. The gene set for the GSEA analysis was sourced from the annotated gene sets available in the Molecular Signatures Database (MsigDB).

### Consensus clustering analysis

A total of 200 Hypoxia-Related Genes (HRGs) obtained from the MSigDB database were employed to identify different hypoxia-related patterns (https://www.gsea-msigdb.org/gsea/msigdb/). Consensus clustering was applied to discover subgroups of CRSwNP associated with the expression of these HRGs, utilizing the κ-means method. The maximum number of categories evaluated was set at nine, with 1000 iterations for each κ. Euclidean distance was selected as the clustering metric.^15^ The number of clusters was determined through the consensus clustering algorithm implemented in the “ConsensusClusterPlus (1.60.0)” *R*-package. The optimal number of clusters was identified using cumulative distribution function curves of consistency scores and heat map features derived from the consistency matrix.

### Machine learning to screen HRGs associated hub genes

The LASSO regression and Random Forest algorithms were employed to identify potential candidate genes for diagnosing CRSwNP. LASSO is a linear model designed for feature selection and regression analysis. By incorporating a penalty function, it allows the coefficients of relatively unimportant variables to be reduced to zero, thereby excluding them from the model. An additional Random Forest model was developed using the “randomForest” package in *R* to assess the importance of genes by evaluating their contribution to predictive accuracy. Genes with an importance value greater than 1 were identified as potential genes for future model development. The hub genes were determined by taking the intersection of results obtained from LASSO regression and the Random Forest analysis.

### Immune infiltration analysis

The single-sample Gene Set Enrichment Analysis (ssGSEA) technique is a computational method employed to assess the activation of biological pathways in individual samples, rather than making comparisons between groups. This method was used to quantify immune cell infiltration and function in each sample utilizing the “GSVA” *R*-package.^16^ Furthermore, correlation analyses were carried out aiming to investigate the relevance between gene expression and immune cell infiltration.

### qRT-PCR

Total RNA was extracted from nasal polyp tissue and normal nasal mucosa samples using the TRIzol method, followed by reverse transcription to obtain complementary DNA (cDNA) using the NovoScript® Plus All-in-One 1st Strand cDNA Synthesis SuperMix, followed by qPCR using NovoStart® SYBR qPCR SuperMix Plus to validate the mRNA expression levels of CXCR4, HMOX1, DTNA, and FBP1. The thermal cycling parameters were 95 °C for 30 s, followed by 40 cycles of 95 °C for 15 s and 60 °C for 30 s, and finally 60 °C for 60 s to 95 °C for 15 s. The relative expression levels of each gene were calculated using the 2^−ΔΔCT^ method, with GAPDH serving as the reference gene for normalization. The primers were presented in Table 1.

**Table 1:** Sequences of primers used in qRT-PCR.

Gene	Forward Primer (5′‒3′)	Reverse Primer (5′‒3′)
CXCR4	AGTCTGGACCGCTACCTGG	GCAAAGATGAAGTCGGGAATA
DTNA	TGCGAATAAGCAGCAAAGG	TTGTTCATGCTCTAGCCGAAG
FBP1	CTCTATGGCATTGCTGGTTCT	TTCCACTATGATGGCGTGTTTA
HMOX1	TTGGCTGGCTTCCTTACCG	GCAGGGCTGATCCCTTCG
GAPDH	GGACTCATGACCACAGTCCA	TCAGCTCAGGGATGACCTTG

### Identification of small-molecule drugs

The Connectivity Map (CMap) is a database that compiles gene expression profiles, utilizing cellular responses to various perturbations to identify possible functional links among treatments, genes, and diseases.^17^ In this study, we conducted a CMap analysis to identify small-molecule compounds aimed at various hypoxia-related subtypes. We retrieved drug signatures from the Connectivity Map database (CMap, https://clue.io/). Our input data included the 100 most significantly up-regulated and 100 most significantly down-regulated genes across the two subtypes. A lower standardized drug score indicates a better potential therapeutic effect of the drug.

## Results

### Expression profile of HRGs in CRSwNP

**Fig. 1:**
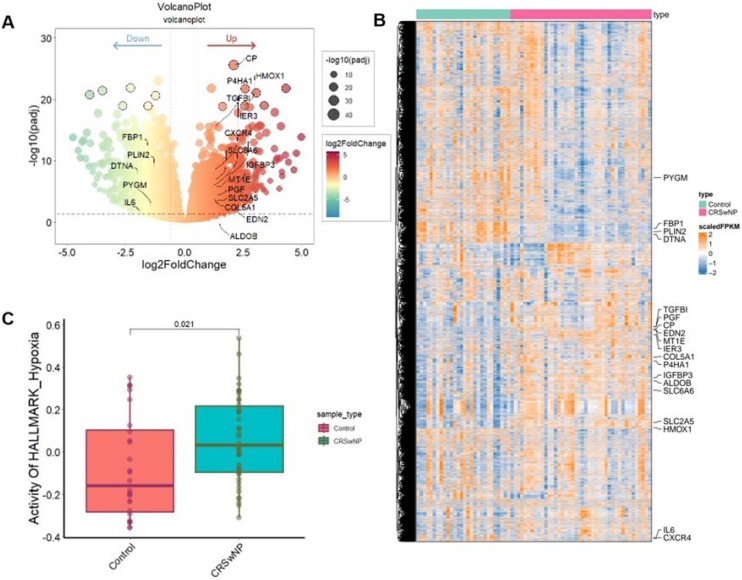
HRGs expressed in control and CRSwNP group. (A) Volcano plots of the DEGs between the control and CRSwNP, and 19 HRG-DEGs are labed. (B) The expression patterns of DEGs were presented in the heatmap, and 19 HRG-DEGs are labed. (C) GSVA analysis of hypoxia activity score between the control and CRSwNP groups. HRGs, Hypoxia-Related Genes; CRSwNP, Chronic Rhinosinusitis with Nasal Polyps; DEGs, Differentially expressed genes; HRG-DEGs, Hypoxia-related differentially expressed genes.

**Fig. 2:**
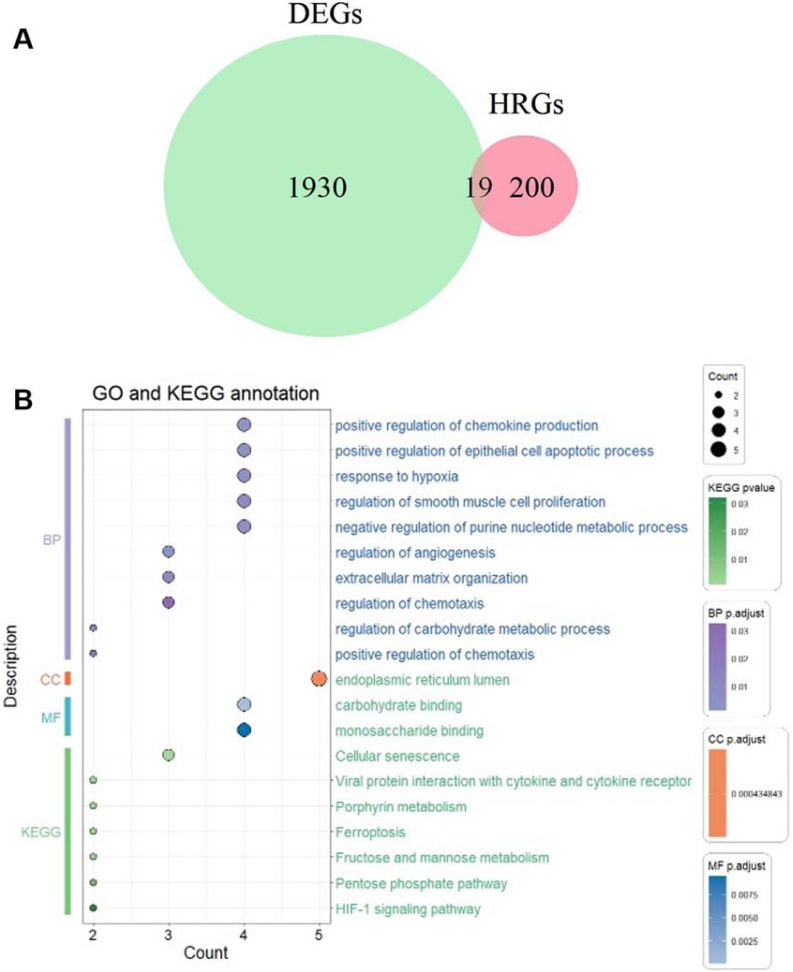
Functional enrichment analysis. (A) The Venn diagram illustrates the distribution of DEGs and HRGs. (B) The dot plot displays the results of GO and KEGG enrichment analyses. DEGs, Differentially Expressed Genes; HRGs, Hypoxia-Related Genes; GO, Gene Ontology; KEGG, Kyoto Encyclopedia of Genes and Genomes.

We analyzed expression profiling data obtained from 28 samples of healthy controls and 42 samples from patients with CRSwNP. Our analysis revealed a total of 1930 DEGs between the CRSwNP and control groups, with 1111 genes upregulated and 819 genes downregulated in the CRSwNP samples (Fig. 1A). A total of 19 HRG-DEGs were identified (Fig. 2A). The heatmap and volcano plot illustrate the distribution of HRG-DEGs. Among these, 14 genes ‒ namely, ALDOB, COL5A1, CP, CXCR4, EDN2, HMOX1, IER3, IGFBP3, MT1E, P4HA1, PGF, SLC2A5, SLC6A6, and TGFBI ‒ were upregulated, while 5 genes ‒ DTNA, FBP1, IL6, PLIN2, and PYGM ‒ were downregulated (Fig. 1A‒B). Furthermore, pathway scores for hypoxia were calculated between the control and CRSwNP groups using the GSVA algorithm. The results demonstrated higher hypoxia pathway scores in the CRSwNP group compared to the control group, highlighting the potential role of hypoxia in CRSwNP pathogenesis (Fig. 1C).

### Functional enrichment analysis

Subsequent GO and KEGG pathway enrichment analysis was conducted on the 19 HRG-DEGs. The GO analysis of Biological Processes (BP) primarily emphasizes pathways such as the positive regulation of chemokine production, response to hypoxia, and regulation of smooth muscle cell proliferation. The Cellular Component (CC) analysis highlights the enrichment of the “endoplasmic reticulum lumen”. Meanwhile, the Molecular Function (MF) annotations focus on various binding activities, including carbohydrate binding and monosaccharide binding. The KEGG pathway analysis (green) reveals significant involvement in metabolic and signaling pathways, including cellular senescence, ferroptosis, fructose and mannose metabolism, and the HIF-1 signaling pathway (Fig. 2B).

### Employing machine learning algorithms to screen hub genes

**Fig. 3:**
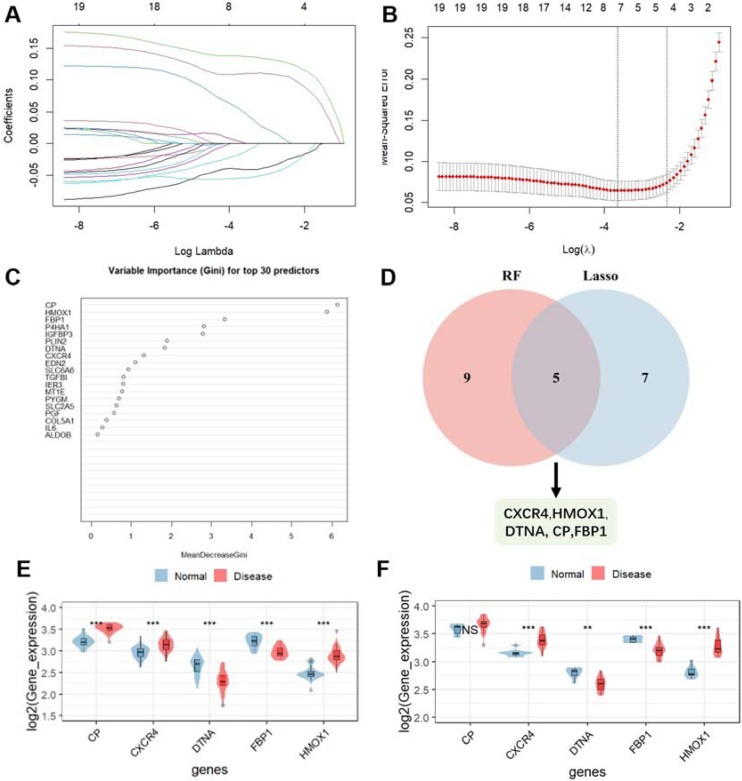
Machine learning models were used to identify core genes (A‒B) LASSO regression analysis was used to select the genes. (C) The genes were selected based on the random forest model. (D) Five key hypoxia-related genes were identified from the intersection of two machine learning algorithms. (E‒F) The expression levels of CP, CXCR4, HMOX1, DTNA, and FBP1 in the test (GSE136825) and validation cohorts (GSE179265). LASSO, Least Absolute Shrinkage and Selection Operator.

We conducted an intersection analysis among the genes identified by two machine learning algorithms to screen hub genes. LASSO regression analysis revealed seven feature genes (CP, CXCR4, DTNA, FBP1, HMOX1, IL6, MT1E), as illustrated in Fig. 3A and B. The Random Forest algorithm identified a set of nine feature genes with importance scores exceeding 1 (Fig. 3C). By intersecting the feature genes from the LASSO regression with those from the Random Forest analysis, we derived a list of five overlapping genes: CP, CXCR4, HMOX1, DTNA, and FBP1 (Fig. 3D).

### Validation of hub genes

To further validate the five hub genes identified through machine learning, a GEO dataset (GSE179265) containing bulk RNA-seq data was selected to assess the expression of these hub genes. Consistent with previous analysis, two genes (CXCR4 and HMOX1) were found to be upregulated, while two genes (DTNA and FBP1) were downregulated. However, the expression of the CP gene showed no significant difference (Fig. 3E‒F).

**Fig. 4:**
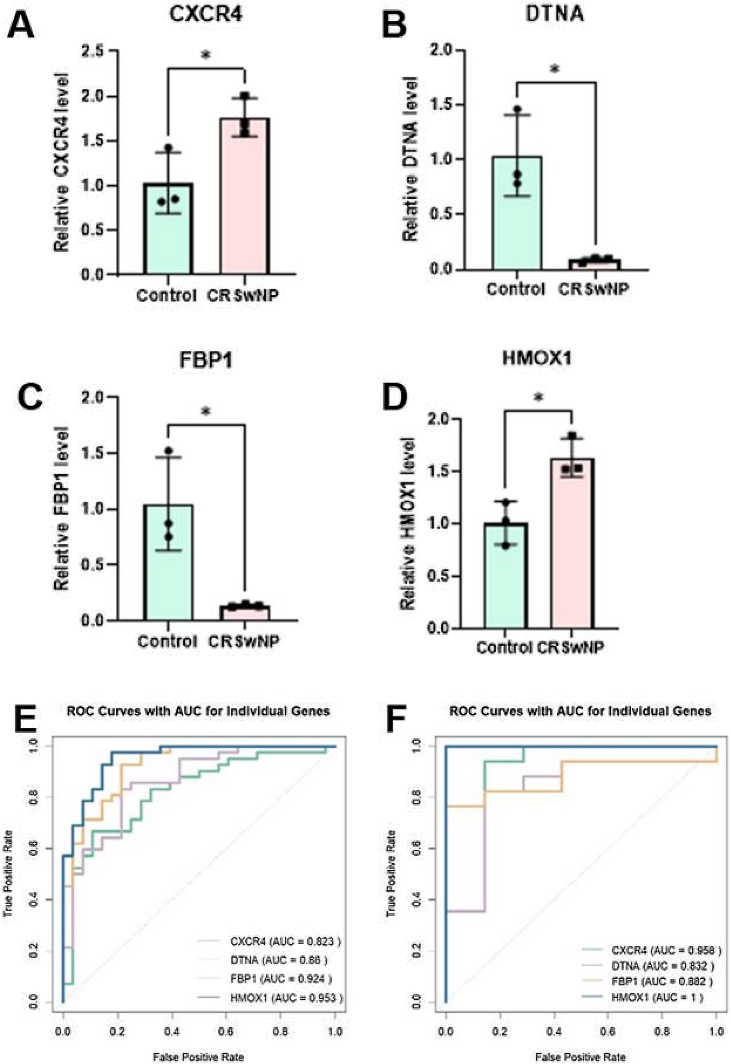
The expression levels of (A) CXCR4, (B) DTNA, (C) FBP1 and (D) HMOX1 in clinical samples, *p < 0.05. (E‒F) ROC curves for evaluating the diagnostic values of CXCR4, DTNA, FBP1 and HMOX1 in the test (GSE136825) and validation cohorts (GSE179265). ROC, Receiver Operating Characteristic curves.

Thus, the CP gene was excluded. Clinical tissue samples from healthy individuals and patients with CRSwNP were also collected to examine gene expression levels using qRT-PCR. The results indicated upregulation of CXCR4 and HMOX1 and downregulation of DTNA and FBP1 in the CRSwNP group compared to the control group (Fig. 4A‒D).

Furthermore, the diagnostic value of these genes was assessed through ROC curve analysis. The areas under the AUC curve for CXCR4, HMOX1, DTNA, and FBP1 were all greater than 0.8 (Fig. 4E‒F). These findings suggest that CXCR4, HMOX1, DTNA, and FBP1 may serve as potential novel diagnostic biomarkers for CRSwNP.

### Cluster analysis

**Fig. 5:**
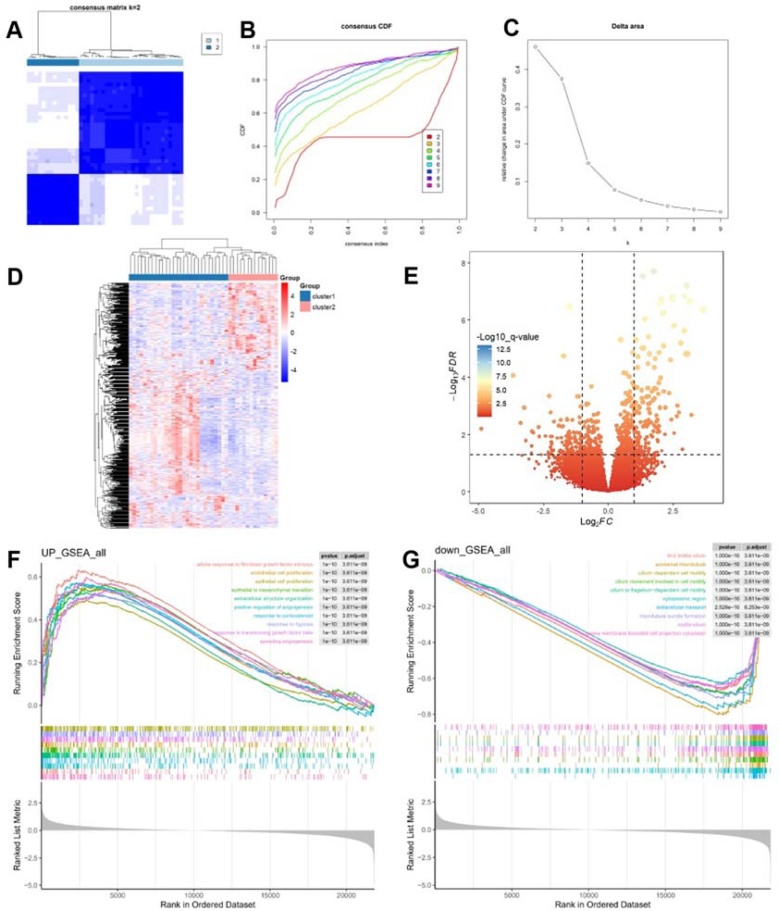
Identification of hypoxia-related subtypes and comprehensive pathway enrichment analysis in CRSwNP. (A) Consensus clustering matrixes for values of κ = 2. (B) CDF curves displayed consensus distributions from κ = 2 to κ = 10. (C) Area fraction under the CDF curve for κ = 2–10. (D‒E) The heamap and volcano map between cluster 1 and cluster 2. (F) Representative upregulated GO gene sets. (G) Representative downregulated GO gene sets. CRSwNP, Chronic Rhinosinusitis with Nasal Polyps; CDF, Cumulative Distribution Function; GO, Gene Ontology.

A consensus cluster analysis of NP samples was conducted to classify patients into different phenotypes using unsupervised cluster analysis based on assessing expression discrepancy of 200 HRGs. The result revealed a clearer distinction between the two sample clusters at κ = 2, leading to the establishment of two distinct clusters (Fig. 5A‒C). DEGs between Cluster 1 and Cluster 2 were also identified. The volcano plot and heatmap depict the distribution of DEGs between the two clusters, revealing a total of 226 upregulated genes and 453 downregulated genes in Cluster 2 compared to Cluster 1 (Fig. 5D‒E).

### Functional enrichment between clusters

In our GO enrichment analysis using Gene Set Enrichment Analysis (GSEA), pathways that showed significant upregulation and downregulation were enriched. Upregulated pathways include pathological processes related to conditions such as sprouting angiogenesis, epithelial to mesenchymal transition, and cellular responses to fibroblast growth factor stimulus and transforming growth factor beta. The treatment-related pathway ‒ response to corticosteroids ‒ was also upregulated (Fig. 5F). Meanwhile, several pathways were significantly downregulated, indicating potential impairments in cellular functions (Fig. 5G). The pathways related to cilium movement, axoneme assembly, and cilium-dependent cell motility suggest a decreased capability for effective cellular motility. Additionally, the downregulation of epithelial cilium movement involved in extracellular fluid movement points to potential disruptions in fluid homeostasis and epithelial function.

### The outcomes of the infiltration of immune cells

**Fig. 6:**
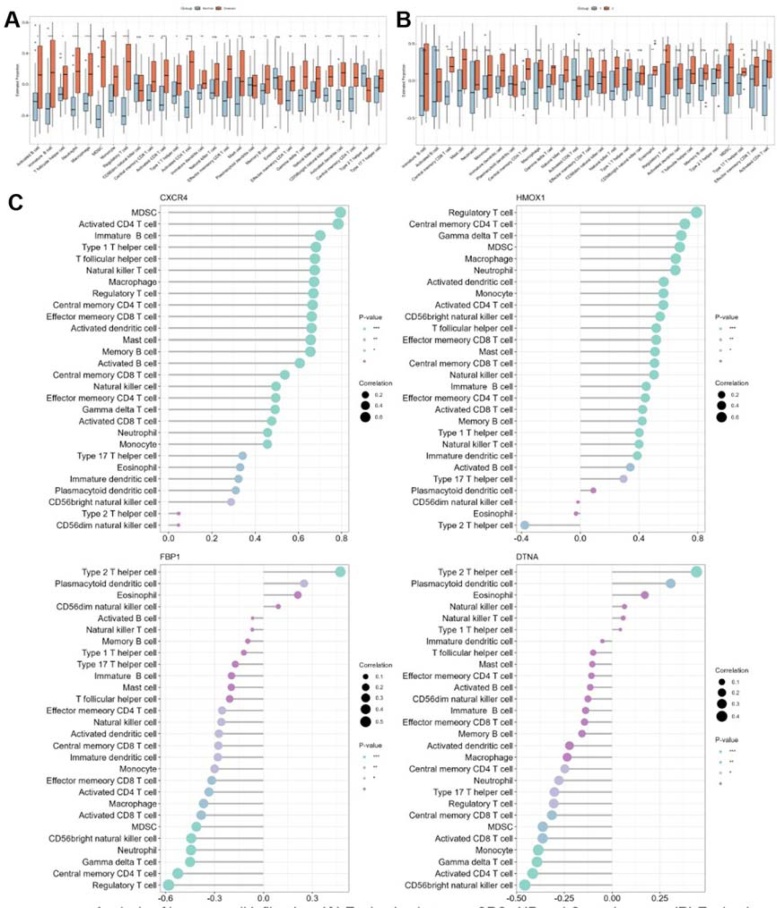
Analysis of immune cell infiltration. (A) Evaluation between CRSwNP and Control groups. (B) Evaluation between cluster 1 and cluster 2. (C) Assessment of the correlation between immune cells and the expression of CXCR4, HMOX1, FBP1 and DTNA. *p < 0.05, **p < 0.01, ***p < 0.001. CRSwNP, Chronic Rhinosinusitis with Nasal Polyps.

To investigate the immune microenvironment of CRSwNP, we employed the ssGSEA algorithm to quantify the relative scores of 28 immune cell types. Comparing these scores between the healthy and CRSwNP groups revealed significant statistical differences in various immune cells, including activated B-cells, CD4 T-cells, CD8 T-cells, dendritic cells, central memory CD4 T-cells, central memory CD8 T-cells, effector memory CD4 T-cells, effector memory CD8 T-cells, and Type 1, Type 2, and Type 17 T-helper cells (Fig. 6A). Notably, CRSwNP samples exhibited significantly higher levels of immune infiltration compared to control samples.

Furthermore, we investigated the correlation between hub genes and immune cells and found that the hub genes FBP1 and DTNA exhibited a significant negative correlation with most immune cells. In contrast, the hub gene HMOX1 and CXCR4 demonstrated a significant positive correlation with most immune cells (Fig. 6C). The analysis indicated a close association between the hub genes and the degree of immune cell infiltration, signifying their significant role in the immune microenvironment of the disease. Further research is needed to validate these findings and explore the potential clinical applications in the management of CRSwNP.

We also compared immune cell infiltration between Cluster 1 and Cluster 2 to explore the potential reasons for the differences in enriched pathways observed between the clusters. Statistical disparities were noted in various immune cell types, including type 17 T-helper cells, activated CD4 T-cells, eosinophils, and central memory CD4 and CD8 Tcells (Fig. 6B). Notably, Cluster 2 exhibited higher levels of immune infiltration compared to Cluster 1.

### Therapeutic target prediction

Using the CMap database, we analyzed potential therapeutic agents targeting different subtypes of CRSwNP. As illustrated in Table 2, the five small molecules with the highest therapeutic potential in Cluster 1 are talnetant, BRD-K97929312, paroxetine, diphenidol, and BRD-K96612763. In Cluster 2, the five small molecules exhibiting the highest therapeutic potential are alvocidib, lapatinib, U-0126, canertinib, and triptolide.

**Table 2:** CMap analysis identified potential drugs for CRSwNP subtypes.

Subgroups	Rank	Drug name	Standardized scores	Mechanism of drug action
cluster1	1	talnetant	−2.0449	Tachykinin antagonist
	2	BRD-K97929312	−2.0424	/
	3	Paroxetine	−2.0371	Selective serotonin reuptake inhibitor
	4	Diphenidol	−2.0115	Acetylcholine receptor agonist
	5	BRD-K96612763	−2.0087	/
cluster2	1	Alvocidib	−2.5541	CDK inhibitor
	2	Lapatinib	−2.4839	EGFR inhibitor|
	3	U-0126	−2.3495	MEK inhibitor
	4	Canertinib	−2.3132	EGFR inhibitor
	5	Triptolide	−2.2943	/

## Discussion

CRS is a common heterogeneous inflammatory disease of the upper respiratory tract.^6^ Current treatment strategies of CRS are primarily based on phenotypic classifications and often fail to consider the underlying molecular mechanisms and pathophysiology of the disease.^18^ Moreover, previous studies have reported that, even after one year of standard treatment, 33.7% of CRSwNP patients and 20.97% of CRSsNP patients experience refractory or recurrent disease.^8^ These findings highlight the heterogeneity of CRS. The role of hypoxia-related pathological pathways in the development of CRSwNP has been suggested.^10^ To our understanding, this study is the first to investigate and analyze the role of hypoxia in the development of CRSwNP by examining high-throughput sequencing expression data.

In this research, we utilized the GSE136825 dataset to identify HRGs associated with CRSwNP through machine learning techniques. These findings were further validated using the GSE179265 dataset and qRT-PCR analysis of clinical samples. Four HRGs were identified as being significantly linked to CRSwNP: CXCR4, HMOX1, DTNA, and FBP1.

CXCR4 regulates immune cell migration and cytokine production, promoting inflammation through recruitment of immune cells such as neutrophils and macrophages.^19^^,^^20^ Its interaction with CXCL12 supports leukocyte migration, contributing to tissue damage and repair.^21^ While underexplored in sinusitis, its involvement in broader inflammatory diseases suggests a key role in CRSwNP. HMOX1, encoding haem oxygenase-1, provides protection against oxidative stress by inhibiting pro-inflammatory cytokines and promoting inflammation resolution.^22^ Its by-products, like carbon monoxide, further suppress inflammatory pathways, reducing tissue damage.^23^ For FBP1, a study on Hepatocellular Carcinoma (HCC) found that decreased expression of FBP1, a key enzyme in gluconeogenesis, is associated with impaired gluconeogenesis and increased glycolysis.^24^ Metabolites that accumulate under glycolytic conditions have been shown to enhance the inflammatory response by modulating intracellular signaling pathways and altering the epigenetic landscape.^25^ Although FBP1’s role in CRSwNP has been minimally explored, these findings suggest it may influence inflammation through metabolic regulation. Finally, DTNA, encoding α-dystrobrevin, is essential for maintaining cell structure. Downregulation of DTNA may impair cytoskeletal organization and cell adhesion, weakening the epithelial barrier and increasing susceptibility to inflammation and pathogens in the nasal mucosa.^26^^,^^27^

We subsequently conducted consensus clustering analysis on the CRSwNP samples based on the expression profiles of all HRGs, resulting in two distinct clusters. Following enrichment analysis revealed the difference between 2 clusters. Compared to Cluster 1, Cluster 2 displayed significant enrichment and upregulation of pathways associated with sprouting angiogenesis, positive regulation of angiogenesis, and endothelial cell proliferation. This suggests a robust angiogenic response within the affected tissues, indicative of an active remodeling environment.^28^ Additionally, the upregulation of pathways associated with Epithelial-to-Mesenchymal Transition (EMT) and Transforming Growth Factor-beta (TGF-β) receptor signaling indicates significant cellular adaptations occurring within the tissue and the transition from a stable epithelial phenotype to a more plastic mesenchymal phenotype, which can contribute to tissue remodeling and chronic inflammation characteristic of this disease.^29^ Furthermore, our immune infiltration analysis revealed that Cluster 2 exhibited higher levels of immune infiltration compared to Cluster 1, with an increased presence of eosinophils, central memory CD4 T-cells, CD8 T-cells, and other immune cells. Previous research suggests that patients with elevated eosinophil counts in nasal polyps, especially those with eosinophilic sinusitis, tend to respond more favorably to transnasal corticosteroid therapy.^30^ This could potentially explain the observed upregulation of the “response to glucocorticoid” pathway in Cluster 2.

In two clusters, varying degrees of activation of pathological pathways and different levels of immune infiltration were observed, suggesting that patients with CRSwNP could potentially be categorized into distinct subtypes based on HRGs. Furthermore, sensitive drugs for these subtypes were identified through differential gene expression screening. The identification and characterization of these inflammatory subtypes may hold significant implications for the development of personalized therapies for CRSwNP patients.

Our study has several limitations. Firstly, we lacked relevant clinical characteristic data of the patients, such as age, gender, and disease duration, which may hinder our ability to identify important factors contributing to disease progression. Additionally, increasing the sample size of patients is necessary to enhance the robustness of our findings. Furthermore, it is imperative to incorporate both in vivo and in vitro experiments to validate the therapeutic potential of the screened drugs.

## Conclusions

In conclusion, our study investigated the expression differences of hypoxia-related genes between CRSwNP and control samples, identifying four disease-associated genes: CXCR4, HMOX1, DTNA, and FBP1. Additionally, consensus cluster analysis based on HRGs, along with further enrichment analyses of various pathological and treatment-related pathways, revealed two distinct clusters of disease entities. This research provides valuable insights into the potential underlying mechanisms of CRSwNP and underscores the importance of further studies to explore targeted therapeutic strategies for these distinct disease subtypes.

## ORCID IDs

Lulu Song: 0009-0004-0392-4572

Shican Zhou: 0000-0002-2099-4394

Lili Wang: 0009-0000-1787-8993

Ju Lai: 0009-0009-9259-9690

Shiwang Tan: 0000-0002-4031-0842

Chunyan Yao: 0009-0009-0930-352X

Shaoqing Yu: 0000-0003-2208-4535

## CRediT authorship contribution statement

Lulu Song: Original draft, writing. Lili Wang and Shican Zhou: Review and editing. Ju Lai: Validation, Shiwang Tan and Shaoqing Yu: Review and editing. Chunyan Yao, Yawen Gao, Bojin Long: Collection of clinical samples.

## Declaration of Generative AI and AI-assisted technologies in the writing process

During the preparation of this work the author(s) used ChatGTP in order to improve language. After using this tool/service, the author(s) reviewed and edited the content as needed and take(s) full responsibility for the content of the publication.

## Funding

This work was supported by National Key R&D Program of China (2022YFC2504100); National Science Foundation of Shanghai (nº 23ZR1458000); Shanghai Hospital Development Center foundation (nº SHDC12024126); Shanghai General Hospital Integrated Traditional Chinese and Western Medicine (nº ZHYY-ZXYJHZX-202118); and Shanghai Oriental Talent Programme (to Yu).

## Data availability statement

The authors declare that all data are available in repository.

## Declaration of competing interest

The authors declare no conflicts of interest.
